# miRNAs as Biomarkers for Diagnosing and Predicting Survival of Head and Neck Squamous Cell Carcinoma Patients

**DOI:** 10.3390/cancers13163980

**Published:** 2021-08-06

**Authors:** Igor Piotrowski, Xiang Zhu, Tatiana Dandolini Saccon, Sarah Ashiqueali, Augusto Schneider, Allancer Divino de Carvalho Nunes, Sarah Noureddine, Agnieszka Sobecka, Wojciech Barczak, Mateusz Szewczyk, Wojciech Golusiński, Michal M. Masternak, Paweł Golusiński

**Affiliations:** 1 Radiobiology Lab, Department of Medical Physics, Greater Poland Cancer Centre, 61-866 Poznan, Poland; igor.piotrowski@wco.pl (I.P.); agnieszka.sobecka@wco.pl (A.S.); wojciech.barczak@oncology.ox.ac.uk (W.B.); 2 Department of Electroradiology, Poznan University of Medical Sciences, ul. Garbary 15, 61-866 Poznan, Poland; 3 Burnett School of Biomedical Sciences, College of Medicine, University of Central Florida, Orlando, FL 32827, USA; xiang.zhu@ucf.edu (X.Z.); sarah.ashiqueali@knights.ucf.edu (S.A.); anunes@umn.edu (A.D.d.C.N.); sarahnoureddine@knights.ucf.edu (S.N.); Michal.Masternak@ucf.edu (M.M.M.); 4 Centro de Desenvolvimento Tecnológico, Universidade Federal de Pelotas, Pelotas 96010-610, Brazil; tatiana.saccon@ufpel.ucf.edu; 5 Faculdade de Nutrição, Universidade Federal de Pelotas, Pelotas 96010-610, Brazil; augusto.schneider@ufpel.edu.br; 6 Department of Head and Neck Surgery, Poznan University of Medical Sciences, 61-701 Poznan, Poland; mateusz.szewczyk@wco.pl (M.S.); wojciech.golusinski@wco.pl (W.G.); 7 Department of Head and Neck Surgery, Greater Poland Cancer Centre, 61-866 Poznan, Poland; 8 Department of Otolaryngology and Maxillofacial Surgery, University of Zielona Gora, 65-417 Zielona Gora, Poland; 9 Department of Maxillofacial Surgery, Poznan University of Medical Sciences, 61-701 Poznan, Poland

**Keywords:** head and neck squamous cell carcinoma, oral cancer, oropharyngeal cancer, laryngeal cancer, miRNA

## Abstract

**Simple Summary:**

Head and Neck Squamous Cell Carcinoma (HNSCC) is the sixth most common cancer worldwide. It arises from the epithelium of the upper aerodigestive tract. Increasing evidence suggests that there is a significant role of microRNAs in HNSCC formation and progression. The aim of this study was to explore and compare the expression of HNSCC related miRNAs in tumor vs neighboring healthy tissue of HNSCC patients with tumors located in either the oral cavity, oropharynx, or larynx. Our results demonstrated that expression of these miRNAs was significantly different not only between healthy and tumor tissues, but also among tumor locations. Further analysis indicated that microRNA expression could be used to distinguish between tumor and healthy tissues, and prognose the overall survival of patients.

**Abstract:**

Head and Neck Squamous Cell Carcinoma (HNSCC) is the sixth most common cancer worldwide. These tumors originate from epithelial cells of the upper aerodigestive tract. HNSCC tumors in different regions can have significantly different molecular characteristics. While many microRNAs (miRNAs) have been found to be involved in the regulation of the carcinogenesis and pathogenesis of HNSCC, new HNSCC related miRNAs are still being discovered. The aim of this study was to explore potential miRNA biomarkers that can be used to diagnose HNSCC and prognose survival of HNSCC patients. For this purpose, we chose a panel of 12 miRNAs: miR-146a-5p, miR-449a, miR-126-5p, miR-34a-5p, miR-34b-5p, miR-34c-5p, miR-217-5p, miR-378c, miR-6510-3p, miR-96-5p, miR-149-5p, and miR-133a-5p. Expression of these miRNAs was measured in tumor tissue and neighboring healthy tissue collected from patients diagnosed with HNSCC (n = 79) in either the oral cavity, oropharynx, or larynx. We observed a pattern of differentially expressed miRNAs at each of these cancer locations. Our study showed that some of these miRNAs, separately or in combination, could serve as biomarkers distinguishing between healthy and tumor tissue, and their expression correlated with patients’ overall survival.

## 1. Introduction

Head and Neck Squamous Cell Carcinoma (HNSCC) accounts for approximately 830,000 new cases and 430,000 deaths annually, making it the sixth most common cancer worldwide [1]. HNSCC includes tumors located in the oral cavity, larynx, nasopharynx, oropharynx, and hypopharynx, contributing to the heterogeneous nature of this disease. The most commonly recognized risk factors for oral and laryngeal cancer include tobacco use and alcohol consumption, whereas the oropharyngeal tumors are often linked to infection by human papillomavirus (HPV). Although some progress has been made in the diagnosis and therapy of HNSCC, the 5-year survival of these patients over the last couple of decades has improved modestly compared to the survival of patients diagnosed with other solid tumors [2]. Malignant transformation in HNSCC begins with hyperplasia of mucosal epithelial cells followed by dysplasia, formation of carcinoma in situ, and finally invasive carcinoma. Recognition of molecular markers that are responsible for carcinogenesis and progression of the disease is crucial for enhanced understanding of the mechanisms behind these processes and could assist in improved diagnosis and prediction of patients’ outcomes.

MicroRNAs (miRNA) are non-coding, short (around 22 nucleotides in length), RNA molecules that regulate the expression of genes through binding to mRNAs, ultimately targeting them for degradation [3]. miRNAs are shown to regulate over 60% of human protein-coding genes [4], and are active in many physiological processes, including apoptosis, aging, and the cell cycle [5,6], as well as in the development of many pathological processes [7]. miRNAs also play an important role in carcinogenesis. Some research shows that miRNA expression profile in solid tumors is significantly altered from that of healthy tissue [8]. Since miRNAs are stably expressed in human tissues and are linked with the initiation and progression of cancer, they could serve as biomarkers for diagnosis, prediction of response to therapy and treatment outcome [9]. Research on HNSCC indicated that many miRNAs are differentially expressed in cancer tissue, with some classified as oncogenic, and others as tumor suppressors [10]. Although the roles of miRNAs in cancer are widely studied, novel cancer-related miRNAs are still being identified [11].

Since miRNAs play a crucial role in cancer development, many studies have investigated the application of miRNA expression analysis as a prognostic tool for HNSCC. Recently published data showed that expression of miRNAs in healthy and tumor tissue of oral squamous cell carcinoma can be associated with patient prognosis, and could be used to predict recurrence and patient survival [9,12]. Several studies demonstrated that expression of miRNAs in patients’ serum could be used as a factor predicting patients’ survival, disease progression, or risk of developing side effects [13,14,15]. Some researchers also pointed out that prognostic, cancer-related miRNAs present in the bloodstream likely originate from tumor cells [14,16]. Identifying a miRNA expression profile in HNSCC tumor is of remarkable importance for establishing pathways of disease development and progression, which could help to elucidate potential targets in novel therapeutic approaches.

To achieve this objective, we investigated the differential expression of miRNAs in HNSCC, analyzed the potential of these miRNAs as biomarkers distinguishing between healthy and tumor tissue, and correlated their expression with patient survival. We assessed matched tumor and neighboring healthy tissues collected from 79 patients with either oral cavity, oropharynx, or larynx HNSCC and we investigated 12 miRNAs of interest: miR-146a-5p, miR-449a, miR-126-5p, miR-34a-5p, miR-34b-5p, miR-34c-5p, miR-217-5p, miR-378c, miR-6510-3p, miR-96-5p, miR-149-5p, and miR-133a-5p. The panel was selected based on a literature search of their differential expression in HNSCC tissue [11,16,17,18,19,20,21,22,23]. Among them, miR-378c and miR-6510-3p were identified in our previous study via analysis of the Cancer Genome Atlas (TCGA) dataset of HNSCC cases [11]. The miRNA panel was chosen to include both miRNAs with oncogenic (i.e., miR-96-5p) and tumor suppressor (i.e., miR-217-5p) activity, as both types of miRNA can be associated with disease progression and outcome [20,21]. Although the miRNAs chosen for analysis were previously shown to play a role in HNSCC, their expression in tumor and healthy tissue in different HNSCC locations and association with patient survival have not been fully investigated.

## 2. Materials and Methods

### 2.1. Study Subjects

In this study, we recruited a total of 79 patients diagnosed with head and neck squamous cell carcinoma in either the oral cavity (*n* = 37), oropharynx (*n* = 9), or larynx (*n* = 33), between 2015 and 2017 at the Department of Head and Neck Surgery at The Greater Poland Cancer Centre in Poznan, Poland. Table 1 presents patients’ characteristics. Staging was determined based on the TNM 7th edition by The Union for International Cancer Control (UICC). Exclusion criteria for participation in the study was defined as follows: patients diagnosed with more than one malignancy, patients previously undergoing other therapeutic modalities such as chemo- and radiotherapy, and HPV positive malignancies. The study protocol was approved by the Ethics Committee of the Poznan University of Medical Sciences (Decision No. 915/16), and written informed consent was provided by all participants.

### 2.2. Experimental Methods

Patients were subjected to primary surgical treatment, during which cancer tissue as well as normal epithelium tissue (within 2 cm distance from the tumor margin) were collected. Both tissues were placed in cryovials on dry-ice directly following the surgical excision and subsequently transferred to −80 °C freezer, and later to liquid nitrogen for long-term storage.

The frozen tissue samples were homogenized with a mortar and pestle with Qiazol (Qiagen, Valencia, CA, USA). Total RNA was isolated using a commercial column purification system (miRNeasy Mini Kit, Qiagen, Valencia, CA, USA) with a DNase treatment (RNase-free DNase Set, Qiagen, Valencia, CA, USA) following manufacturer’s instructions. RNA concentration and purity were measured spectrophotometrically (absorbance at 260, 230, and 280 nm) using Epoch™ Microplate Spectrophotometer (BioTek, Winooski, VT, USA).

To measure miRNA expression, 10 ng of total RNA were reverse-transcribed using TaqMan^®^ Advanced miRNA cDNA Synthesis Kit (Applied Biosystems, Foster City, CA, USA), according to the manufacturer’s instructions. For the RT-qPCR reaction a 1:10 dilution of cDNA template was prepared. RT-qPCR was performed using the Quantstudio™ 7 Flex System. We used TaqMan^®^ Advanced miRNA Assays (Applied Biosystems, Foster City, CA, USA) with primers specific for selected miRNA: miR-146a-5p (478399_mir), miR-449a-5p (478561_mir), miR-126-5p (477888_mir), miR-34a-5p (478048_mir), miR-34b-5p (478050_mir), miR-34c-5p (478052_mir), miR-217-5p (478773_mir), miR-378c-5p (478864_mir), miR-6510-3p (480744_mir), miR-96-5p (478215_mir), miR-149-5p (477917_mir), miR-133a-5p (478706_mir). Supplemental Table S1 presents stem loops used for miRNA expression analysis. Each reaction was performed in two technical replicates. The 2^−∆∆Ct^ method was used to calculate and normalize expression of each miRNA, in which −∆∆Ct was calculated as:

−∆∆Ct = average (∆Ct _healthy tissue_) − ∆Ct _tumor tissue,_ (1)

where

∆Ct = Ct _target gene_ − Ct _housekeeping gene._ (2)

The housekeeping gene was miR-16-5p. In the above calculation, −∆∆Ct > 0 denotes upregulated expression, whereas −∆∆Ct < 0 denotes downregulated expression, relative to healthy tissue.

### 2.3. Statistical Analysis

**Figure 1:**
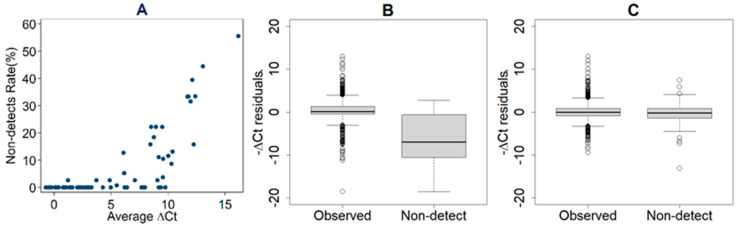
The scatter plot (**A**) indicating that the non-detect rate increases with average ΔCt by location, tissue type and gene. The boxplots showing the comparison of −ΔCt residues between observed expressions and non-detects, (**B**) if conventional imputation of setting the Ct of each non-detect to 40 was conducted, and (**C**) if expectation-maximization (EM) algorithm was used to estimate the Ct of each non-detect.

Through RT-qPCR experiments, we obtained 1785 expression data points with an observed Ct of <40 and 135 non-detects without an assigned Ct value. We noticed that the non-detect rate in our RT-qPCR experiment increased with average ΔCt of each gene (Figure 1A), suggesting that the non-detects are not randomly distributed and imputation must be implemented. We first evaluated the bias of the non-detects imputed by a conventional method of setting the Ct of each non-detect to 40. The results showed that the bias caused by such an imputation method remarkably skewed the data towards low expression (Figure 1B). To deal with this issue and impute the missing expression data more appropriately, we then performed the expectation-maximization (EM) algorithm proposed by McCall et al. [24] using R package (HTqPCR, www.bioconductor.org/packages/release/bioc/html/HTqPCR.html, accessed on 11/16/2020) [25]. The results indicated that the EM algorithm produced unbiased imputation for the non-detects (Figure 1C).

Wilcoxon matched-pairs signed-ranks test was used to compare the expression of each miRNA in tumor tissue vs. in healthy tissue. Heatmap was produced to visualize the expression profile of each miRNA across tumor locations and tissue types. To evaluate the accuracy of each miRNA expression as a biomarker in distinguishing tumor from healthy tissue, we performed bootstrap logistic regression based on which ROC curves were produced, and sensitivity, specificity, and AUC were estimated. In addition, we performed stepwise logistic regression to explore whether combined miRNA expressions can be used as a more valid biomarker in the diagnosis of HNSCC. Finally, we assessed the prognosis value of each miRNA through survival analysis using the Kaplan–Meier survival curves. In this analysis, we first used R package (survminer) to find an optimal cut-off, splitting the expression of each miRNA into low and high expression. We then compared survival curves between low and high expression using logrank test. To further evaluate the prognosis of the selected miRNAs on overall survival of the HNSCC patients, we performed multiple Cox regression analysis with age at diagnosis, T stage, and N stage included as confounding variables. Due to the fact that the results of the small cohort of oropharyngeal cancer (n = 9) might be prone to bias, they were only presented in Supplementary Figures. Statistical analysis was performed using Stata MP software 14.1 (StataCorp LLC, College Station, TX, USA) and R packages. All tests were two-tailed and the *p* value < 0.05 was considered to be statistically significant.

## 3. Results

### 3.1. miRNA Expression

**Figure 2:**
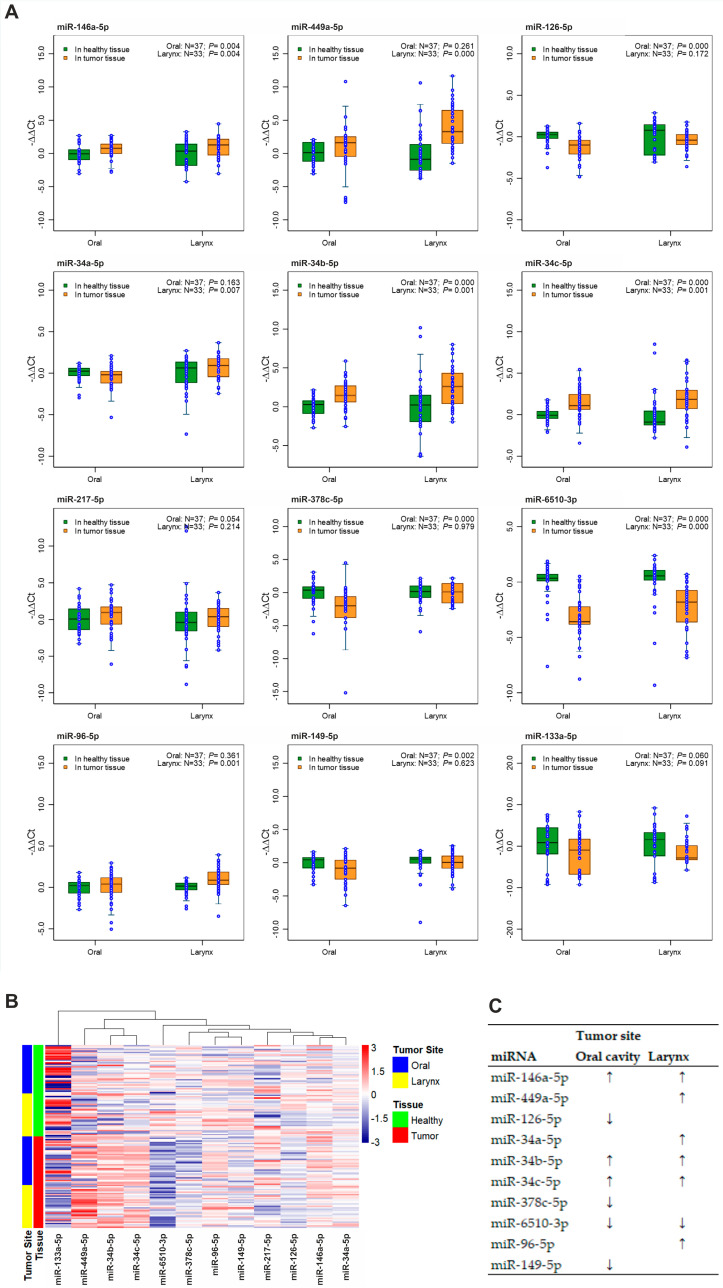
Expression of miRNAs in tumor tissue and nearby healthy tissue of HNSCC patients. (**A**) Boxplots showing the comparison of expression of 12 miRNAs between tumor tissue and nearby healthy tissue in the oral cavity, and larynx. Expression was calculated using −∆∆Ct method; values above 0 denote upregulation, and values below 0 denote downregulation of miRNA, compared with healthy tissue. Wilcoxon signed-rank test was performed. (**B**) Heatmap and dendrogram showing the overall expression profile of the 12 miRNAs in patient samples. Samples were grouped by tissue type and tumor location. The colors in the heatmap represent the value of −∆∆Ct ranging from downregulation (dark blue) to upregulation (red). (**C**) Summary of findings on miRNA expressions as biomarkers in diagnosis of HNSCC (“↓” denotes downregulation and “↑” denotes upregulation).

First, we measured the expression of selected miRNAs in tumor tissue and neighboring healthy tissue of HNSCC patients. In oral cancer, we observed upregulation of miR-146a-5p, miR34b-5p, and miR34c-5p, and downregulation of miR-126-5p, miR378c-5p, miR-6510-3p, and miR-149-5p in tumor tissue compared with neighboring healthy tissue (Figure 2A). In laryngeal cancer, we observed upregulation of miR-146a-5p, miR-449a-5p, miR-34a-5p, miR-34b-5p, miR-34c-5p, and miR-96-5p and downregulation of miR-6510-3p (Figure 2A). The median values of miRNA expressions in tumor tissue relative to healthy tissue are presented in supplementary materials (Table S2). Clustering analysis of miRNA expression in each sample revealed that the expression of miRNAs strongly depended on the tissue type (tumor or healthy) and, to a lesser extent, on the tumor location (Figure 2B). Figure 2C presents a simple summary of miRNA expression analysis. The results of miRNA expression analysis for all three tumor locations (including oropharyngeal cancer) are presented in Supplementary Figure S1.

### 3.2. miRNA Expression as a Biomarker to Distinguish Healthy Tissue from Tumor Tissue

#### 3.2.1. Expression of Individual miRNAs as a Biomarker

For the analysis of miRNAs as biomarkers, we considered expression of miRNAs showing both sensitivity and specificity ≥ 0.8 as strong candidates, and miRNAs showing both sensitivity and specificity ≥ 0.7 as potential, weaker candidates. For some miRNA expressions (miR-34c-5p and miR-149-5p in oral cancer, miR-34c-5p in laryngeal cancer) our analysis was not able to determine a unique threshold value, and ROC could not be accurately estimated. 

**Figure 3:**
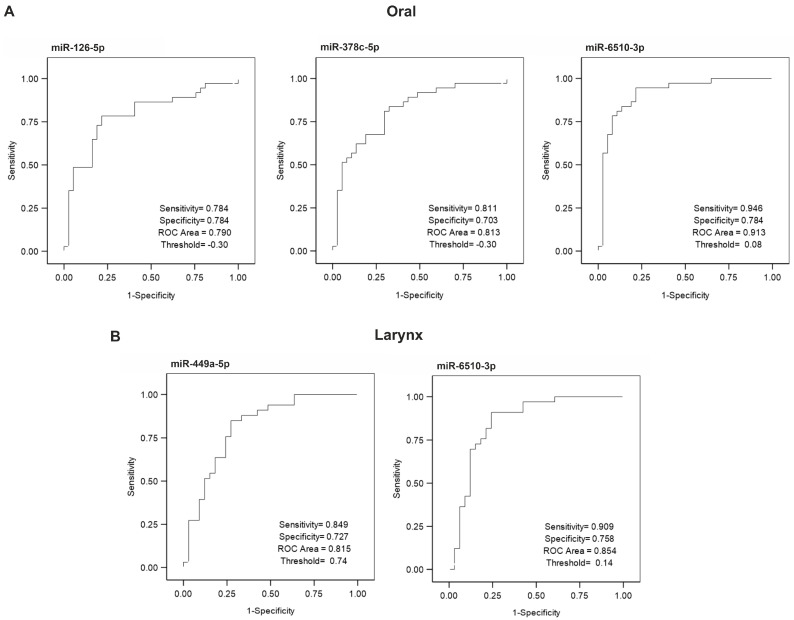
ROC curves showing miRNA biomarkers with potential in distinguishing tumor tissue from nearby healthy tissue in oral (**A**) and laryngeal (**B**) cancer.

For oral cancer, no miRNA expression reached the threshold sensitivity and specificity for a strong biomarker (Figure 3A). Expression of miR-378c-5p (sensitivity = 0.811, specificity = 0.703, ROC Area = 0.813, threshold = −0.3), and miR-6510-3p (sensitivity = 0.946, specificity = 0.784, ROC Area = 0.913, threshold = 0.08) demonstrated satisfying sensitivity and moderate specificity (indicating a higher risk of false positives) as biomarkers. The sensitivity and specificity of the expression of miR-126-5p (sensitivity = 0.784, specificity = 0.784, ROC Area = 0.790, threshold = −0.3) was close to 0.8, suggesting that this miRNA might be a candidate biomarker.

For laryngeal cancer, no miRNA expression reached the threshold sensitivity and specificity for a strong biomarker (Figure 3B). Expression of miR-449a (sensitivity = 0.849, specificity = 0.727, ROC Area = 0.815, threshold = 0.74), and miR-6510-3p (sensitivity = 0.909, specificity = 0.758, ROC Area = 0.854, threshold = 0.14) presented as weaker biomarker candidates, with high sensitivity and moderate specificity (higher risk of false positives). ROC curves for all miRNAs, analyzed in tissues collected form oral, oropharyngeal, and laryngeal cancer, are presented in Supplemental Figures S2–S4. 

#### 3.2.2. Combined Expression of miRNAs as a Biomarker

**Figure 4:**
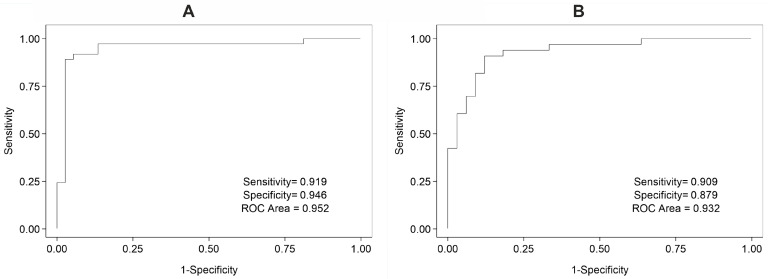
ROC showing accuracy of combined miRNA expression as a biomarker in distinguishing oral (**A**) and laryngeal (**B**) tumor tissue compared with neighboring healthy tissue. (**A**) Combined expression of miR-6510-3p and miR-34c-5p as a biomarker distinguishing oral tumor tissue from nearby healthy tissue with a threshold of −0.194 determined by the best model: logit(tumor) = −2.239 − 1.127(miR-6510-3p) + 1.392(miR-34c-5p). (**B**) Combined expression of miR-449a-5p, miR-6510-3p, and miR-149-5p as a biomarker in distinguishing laryngeal tumor tissue from nearby healthy tissue with a threshold of −0.212 determined by the best model: logit(tumor) = −1.855 + 0.438(miR-449a-5p) − 1.246(miR-6510-3p) + 1.473(miR-149-5p). In both models, if logit(tumor) score is greater than its corresponding threshold, the diagnosis is positive, otherwise the diagnosis is negative.

Since the expression of some miRNAs in neighboring healthy tissue compared to tumor tissue exhibited potential for use as biomarkers distinguishing between the two tissues, we decided to further investigate whether combined miRNA expression could be used with higher success. For oral cancer samples, we observed that the combined expression of miR-6510-3p and miR-34c-5p could be used as a biomarker with high success (sensitivity = 0.919, specificity = 0.946, ROC Area = 0.952) (Figure 4A). Similar analysis was performed for miRNA expression in laryngeal tumor and neighboring healthy tissue, where the combined expression of miR-449a-5p, miR-6510-3p, and miR-133a-5p showed strong ability to distinguish tumor tissues (sensitivity = 0.909, specificity = 0.879, ROC Area = 0.932) (Figure 4B).

### 3.3. Association of miRNA Expressions with Survival of HNSCC Patients

**Figure 5:**
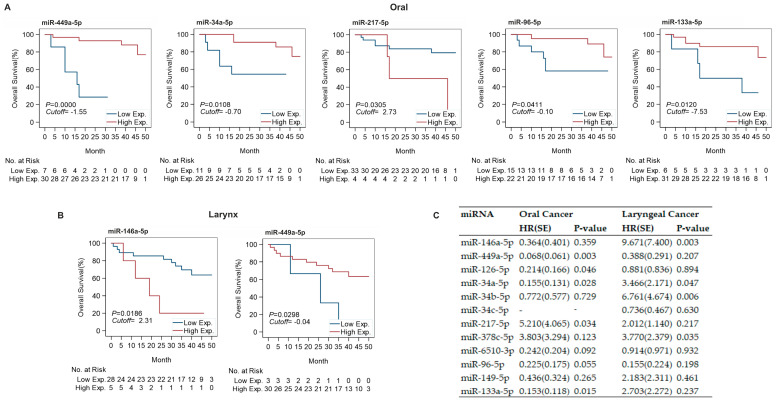
Relation between miRNA expression and overall survival. (**A**,**B**) Kaplan Meier survival curves showing comparison of overall survival between low and high miRNA expression defined by optimal cutoff of −∆∆Ct in oral (**A**) and laryngeal (**B**) cancers. (**C**) Summary of multiple Cox regression analysis for oral and laryngeal cancer investigating hazard ratio (HR) of high expression vs. low expression of each miRNA in separate survival models with age at diagnosis, T staging and N staging under control. HR < 1 denotes high miRNA expression at lower risk compared to low expression. HR > 1 denotes high miRNA expression at higher risk than low expression.

Since expression of some of the selected miRNAs in tumor tissue was significantly different than in neighboring healthy tissue, we proposed that expression of these miRNAs could have a marked impact on patient survival. In oral cancer, low expression of miR-449a-5p (cutoff = −1.55), miR-34a-5p (cutoff = −0.70), miR-217-5p (cutoff = 2.73), miR-96-5p (cutoff = −0.10), and miR-133a-5p (cutoff = −7.53) in tumor tissue correlated with a significantly shorter survival (Figure 5A). Survival of patients with laryngeal cancer was significantly reduced in patients with high expression of miR-146a-5p (cutoff = 2.31) or low expression of miR-449a-5p (cutoff = 0.04) in tumor tissue (Figure 5B). Supplemental Figures S5–S7 present survival data for all miRNAs expressed in the tumor tissues collected from oral, oropharyngeal, and laryngeal cancer.

To verify whether relative miRNA expression in tumor tissue correlated with patient survival, we performed multiple Cox regression analysis, controlling for age at diagnosis, T stage, and N stage (Figure 5C). The analysis indicated that in oral cancer high expression of miR-449a-5p (HR = 0.068), miR-126-5p (HR = 0.214), miR-34a-5p (HR = 0.155), and miR-133a-5p (HR = 0.153) had a lower hazard, corresponding to a higher survival rate, and high expression of miR-217-5p (HR = 5.21) corresponded to a higher hazard and a lower survival rate. In laryngeal cancer, high expression of miR-146a-5p (HR = 9.671), miR-34a-5p (HR = 3.466), miR-34b-5p (HR = 6.761), and miR-378c-5p (HR = 3.77) was associated with a higher hazard and lower survival rate.

We also conducted multiple Cox regression analysis to investigate whether the combined miRNA expression was associated with patients’ overall survival when controlled for age at diagnosis, T staging, and N staging. No significant association with age or staging was observed (Supplementary Table S3).

## 4. Discussion

Although the role of miRNAs in HNSCC development has been widely studied, and new miRNAs with significant oncogenic or cancer suppressor roles are being discovered, expression of these miRNAs in tumor and healthy tissue of patients with HNSCC at different sites has rarely been addressed. We investigated whether the differences in expression of miRNAs can be used as markers to distinguish between tumor and healthy tissue, and whether miRNA expression correlates with patient survival. Based on a literature search for miRNAs differentially expressed in HNSCC tissue [11,16,17,18,19,20,21,22,23], and on our previous study using deep sequencing on small cohorts of HNSCC patients [11], we selected a panel of 12 miRNAs for analysis: miR-146a-5p, miR-449a-5p, miR-126-5p, miR-34a-5p, miR-34b-5p, miR-34c-5p, miR-217-5p, miR-146a-5p, miR-378c-5p, miR-6510-3p, miR-96-5p, miR-149-5p, and miR-133a-5p. Expression of these miRNAs was measured in tumor and healthy tissue of 79 patients diagnosed with HNSCC located in either the oral cavity, oropharynx, or larynx.

It is noted that miR-146a-5p has previously been described in several types of cancer, including head and neck cancer [26]. Zhu et al. [27] observed upregulation of miR-146a-5p in oral cancer cell lines compared with normal keratinocyte cell line, and showed that miR-146a-5p inhibits NF-κB1 and reduces apoptotic cell death in oral cancer cell lines. Recent results show that expression of this miRNA in HNSCC is also connected with the status of human papillomavirus (HPV) infection [28]. Hung et al. also confirmed that this miRNA has an oncogenic and pro-metastatic effect in oral cancer by targeting genes involved in the NF-κB pathway [16]. The same authors also observed a high plasma level of miR-146a-5p in patients with oral cancer compared to controls, which decreased after surgery, suggesting that the miRNA present in plasma originated from the tumor. This indicates that miR-146a-5p might be a useful HNSCC marker in both tumor tissue and circulation. It is important to note that other studies point towards an anti-oncogenic effect of miR-146a-5p related to the targeting of SOX2 in oral cancer cell lines [29]. Only one study connected miR-146a-5p expression with laryngeal cancer and observed an increased risk of this cancer in individuals with the rs2910164 polymorphism in the miR-146a-5p gene [30]. In our study, we showed an upregulation of miR-146a-5p in oral and laryngeal tumor tissue, which was also observed by others [27]. Furthermore, our results indicate that laryngeal cancer patients with high expression of miR-146a-5p show worse overall survival, which is in line with its oncogenic role described in the literature.

It is noted that miR-449a-5p has been identified as a potential anti-oncogenic factor in several cancers [31,32]. Expression of miR-449a-5p was shown to be downregulated in tumor tissue compared with normal tissue in nasopharyngeal carcinoma [17]. Studies show that miR-449a has a suppressive effect on the progression, migration, and sphere formation of nasopharyngeal cancer cells [33,34]. In nasopharyngeal cancer, this miRNA was found to suppress LDH oncogenic activity [33]. Recently a decreased expression of miR-449a-5p was shown to correlate with positive nodal status in laryngeal cancer [35]. Interestingly, in our study, the expression of miR-449a-5p was significantly upregulated in laryngeal tumor tissue compared with healthy tissue. Downregulation of miR-449a-5p in oral and laryngeal tumor tissue compared with healthy tissue also correlated with reduced overall survival. Additionally, miR-449a-5p expression showed potential value as a biomarker for differentiating between healthy and cancerous tissue, with relatively high sensitivity and moderate specificity in laryngeal cancer.

The role of miR-126-5p has been described in different cancer types. Sasahira et al. observed its downregulation in oral tumor tissue relative to normal oral mucosa [18]. The same authors showed that decreased levels of miR-126-5p activate vascular endothelial growth factor (VEGF)-A and, consequently, prompt angiogenesis and lymphangiogenesis. Additionally, low expression of miR-126-5p in tumor tissue correlated with a more advanced stage of the disease, positive nodal status, as well as a poor disease-free survival rate in oral cancer patients. Low expression of miR-126-5p in oral squamous cell carcinoma has also been observed by others, with evidence of negative regulation of tumorigenesis via targeting of KRAS, as well as migration and invasion of cancer cells via downregulation of ADAM9 [36,37]. miR-126-5p was also detected in serum exosomes of oral carcinoma patients, and its level correlated with patient survival [38]. Our analysis showed a markedly downregulated expression of miR-126-5p in tumor tissue compared with healthy tissue in patients with oral tumors. Low expression of miR-126-5p was also associated with a lower survival rate. Interestingly, in esophageal cancer, this relationship might be opposite—patients with low tumor expression of miR-126 were characterized by worse prognosis than patients with high miR-126-5p expression [39]. This suggests that the location of HNSCC tumor might play a crucial role in the effect that miRNA expression has on the therapy outcome. While miR-126-5p is downregulated in tumor tissue compared to healthy tissue, it is upregulated in plasma of patients with oral cancer compared with healthy individuals, suggesting its potential as a biomarker [40]. Our analysis showed that miR-126-5p expression is a moderate biomarker in distinguishing between tumor and healthy tissue in oral cancer.

Similar to miR-126-5p, the dysregulation of miR-34 family expression has been observed in various types of cancer. miRNAs of the miR-34 family, and miR-34a in particular, are directly regulated by p53, which is a tumor suppressor protein [41]. The miR-34 family is involved in cell-cycle regulation, downregulation of epithelial-to-mesenchymal transition (EMT), suppression of stem-like phenotype, induction of apoptosis and senescence, and inhibition of glycolysis [41]. In a comprehensive meta-analysis, Li et al. concluded that miR-34a-5p is generally expressed at lower levels in HNSCC tumor tissue compared with control tissue [42]. Li et al. also suggested that the level of miR-34a measured in tumor tissue might serve as a clinical biomarker of the disease progression. Our data showed an upregulation of miR-34a-5p in laryngeal cancer tissue compared with healthy tissue, and no difference in oral cancer. An upregulation of miR-34a in tumor vs healthy tissue was also observed by Kalfert et al. only in oropharyngeal cancer, and not in laryngeal cancer [43]. Kalfert et al. underscored that the difference in expression of miR-34a supports the hypothesis of site-specific oncogenesis of HNSCC. Additionally, Kalfert et al. showed a positive correlation between p16 positivity, which is a surrogate marker for HPV infection, and miR-34a expression. As only oropharyngeal cancer cases showed p16 positivity, the miR-34a increase in this tumor location might be explained by p16 status. However, it cannot explain the increase in miR-34a-5p observed in our study, since HPV positivity was one of the exclusion criteria. In our study, oral cancer patients with high expression of miR-34a showed significantly better overall survival than patients with low miR-34a expression, which is in line with the tumor suppressor role of this miRNA. For laryngeal cancer the Cox analysis revealed that high expression of miR-34a-5p was also associated with lower survival rate.

Despite having a similar tumor suppressive role to miR-34a, miR-34b has been found to be upregulated in HNSCC tumor tissue compared with neighboring healthy tissue [44]. Roy et al. observed a significant upregulation of miR-34b expression in oral cancer tissue compared with normal tissue, however the normal tissue was collected from distinct healthy patients [45]. A significant upregulation of miR-34b-5p was also observed in both oral and laryngeal cases of HNSCC in our study. Ren et al. showed that expression of miR-34b was significantly downregulated in metastatic oral cancer tissue compared with nonmetastatic tissue, which is consistent with suppressive effect of miR-34b on EMT [46]. Our analysis indicated that in laryngeal cancer patients, higher expression of miR-34b-5p was associated with lower survival rate. 

Both miR-34b and miR-34c are encoded by a common transcript, separate from miR-34a [47]. Severino et al. showed that, similar to miR-34b, miR-34c expression is upregulated in HNSCC tumor tissue compared with tumor-free margins [44]. However, other studies exhibited downregulated expression of this miRNA in laryngeal carcinoma tumor tissue compared with negative margins [48,49]. miR-34c acts as a tumor suppressor, with some studies displaying suppressive effect of this miRNA on cancer growth and invasiveness [48], and others showing significantly worse overall survival in patients with low expression of miR-34c in laryngeal tumor tissue [50]. Our results revealed that miR-34c-5p expression was significantly upregulated in tumor tissue compared with neighboring healthy tissue in both oral and laryngeal tumor tissue.

Research on the role of miR-217-5p in cancer revealed a potential suppressive effect in different cancer types [20]. In esophageal cancer, this miRNA was found to be repressed after exposure of cells to cigarette smoke condensate, which in turn resulted in overexpression of miR-217-5p’s direct target, kallikrein 7 (KLK7), which induced growth and invasiveness of cancer cells [51]. Miao et al. demonstrated that the expression of miR-217 is significantly lower in laryngeal cancer tissue compared with adjacent paracarcinoma tissue, and also confirmed its suppressive effect on cancer migration, invasion, and proliferation, as well as its ability to induce apoptosis and G1 arrest in a laryngeal cancer cell line [20]. Our results indicated no regulation of miR-217-5p in oral or laryngeal cancer tissue compared with healthy tissue. Additionally, our results revealed a tendency of shorter overall survival of oral cancer patients with high expression of miR-217-5p. The inverse correlation of miR-217-5p expression with survival is contradictory to its anti-tumorigenic role.

Although miR-378c-5p has been recognized in some types of cancer, its role was not yet described in HNSCC. In colon cancer, expression of miR-378c is significantly downregulated in tumor tissue compared with normal colorectal mucosa tissue [52]. miR-378c was recognized as a potential early, progressively downregulated marker in carcinogenesis of esophageal carcinoma [53]. Results previously published by our group showed the differential expression of miR-378c in a TCGA dataset of HNSCC cases for the first time [11]. We demonstrated a significant downregulation of miR-378c in HNSCC tumor tissue compared with neighboring healthy tissue. Additionally, expression of miR-378c inversely correlated with the cancer T stage, suggesting a potential role in disease progression. In the current study, we confirmed the downregulation of miR-378c-5p in oral tumor tissue compared with healthy tissue. In laryngeal cancer, no significant regulation was observed. High expression of this miRNA in laryngeal tumor tissue was associated with lower survival rate. Expression of miR-378c-5p also displayed potential as a biomarker differentiating oral tumor tissue from healthy tissue with good sensitivity and moderate specificity.

The data on miR-6510-3p related to cancer are very scarce. Chen et al. found that this miRNA is significantly downregulated in oral lichen planus (OLP) buccal mucosa compared with normal mucosa from healthy individuals [54]. OLP is characterized by a tumor-like microenvironment and is associated with an increased risk of developing oral squamous cell carcinoma [55,56]. The downregulated expression of miR-6510-3p in OLP suggests that it might play a significant role in premalignant transformation in squamous cell carcinoma development. In a previous study, we demonstrated significant downregulation of miR-6510-3p expression in HNSCC tumor tissue compared with healthy tissue, as well as an inverse correlation between miR-6510-3p expression and the cancer T stage [11]. In this study, a strong downregulation of miR-6510-3p in tumor tissue compared to healthy tissue was also observed in both investigated tumor locations. Our study also showed that miR-6510-3p expression in oral and laryngeal cancer could serve as a biomarker distinguishing between healthy and tumor tissue, with very high sensitivity and specificity.

The role of miR-96-5p has previously been recognized in different types of cancer, including HNSCC. Wang et al. determined that this miRNA is significantly upregulated in oral tumor tissue compared with adjacent normal tissue, and confirmed that miR-96-5p induces proliferation, invasion, and EMT in oral cancer cells through reduction in FOXF2 expression [21]. Other studies on oral cancer cell lines suggested that miR-96-5p can induce chemo- and radio-resistance [57]. In our study, the increased expression of miR-96-5p in oral cancer tissue was not significant; however, we did observe a significantly shorter overall survival of patients with low expression of miR-96-5p, which seems contradictory to its pro-oncogenic role proposed by others. We also observed upregulated miR-96-5p expression in laryngeal cancer tissue. These results suggest that the change in expression of this miRNA might depend on the location of HNSCC.

The role of miR-149-5p has been described in several types of cancer including HNSCC [58,59]. The data on miR-149-5p expression in HNSCC tumor tissue suggests potential dependence on tumor location. Recent results indicate that in oral carcinoma cells miR-149-5p targets CDK6, an oncogene that regulates the cell cycle [60]. Tu et al. showed that the level of miR-149 was downregulated in HNSCC tumor tissue compared with non-cancerous tissue matched to the tumor location [22]. Additionally, expression of this miRNA in lymph node metastatic lesion was lower than both matched primary tumor and matched non-cancerous tissue and its low expression correlated with worse survival. This suggests that miR-149-5p might be an important factor in HNSCC progression. Similarly, research on tongue squamous cell carcinoma displayed downregulation of this miRNA in tumor tissue [61]. Interestingly, in oral cancer, the expression of miR-149-5p was significantly upregulated in tumor tissue compared with peripheral control tissue [62]. Our results showed that expression of miR-149-5p was significantly downregulated in oral tumor tissue, but no difference was observed in laryngeal cancer.

It is noted that miR-133a-5p has been recognized as a HNSCC suppressor inducing apoptosis and inhibiting migration of cancer cells [23,63]. Several studies showed downregulation of miR-133a in HNSCC tumor tissue of various locations compared with normal adjacent tissues [63,64]. Recent research indicates that in oral carcinoma miR-133a-5p acts as a tumor suppressor, inducing apoptosis, and inhibiting proliferation, migration and invasion through regulation of Notch signaling pathway [65]. Others indicated that the suppressive effect of this miRNA in laryngeal cancer might be related to the targeting of CD47, a transmembrane protein widely expressed in tumor cells [66]. Our results also indicated a downregulation of miR-133a-5p expression in oral and laryngeal tumor tissue; however, the difference was not significant. The low expression of this miRNA in oral cancer patients also correlated with shorter overall survival. 

We also analyzed whether miRNA combinations could be used as a biomarker. Our analysis allowed us to identify two sets of miRNAs in oral and laryngeal cancer. In oral cancer combined expression of miR-6510-3p and miR-34c-5p showed very high sensitivity and specificity in distinguishing between tumor and healthy tissue. Both miRNAs were previously found to have a suppressive role in HNSCC. In laryngeal cancer, the combined expression of miR-449a-5p, miR-6510-3p, and miR-149-5p presented as a valuable biomarker for distinguishing tumor and healthy tissue. Similar to the oral cancer miRNAs, these three biomarker miRNAs were shown to have tumor suppressive effects in HNSCC. Both sets were better in distinguishing the two tissues than any single miRNA expression separately. This suggests that the combined targets of these miRNAs can potentially accelerate cancer progression and malignancy, decreasing the chances of disease-free survival. However, in this study we were not able to demonstrate a correlation between expression of these miRNA sets and overall survival of patients.

Although our study reveals some new findings regarding the expression of selected miRNAs in tumor and healthy tissue of patients diagnosed with oral, oropharyngeal, or laryngeal cancer, it has some limitations. The analysis of oropharyngeal cancer was based on a small sample of patients (*n* = 9). Although we observed significant differences in miRNA expression in this group, the power to make a correct conclusion about survival and ROC analysis may be limited due to the small sample size. In the future, further investigations in a larger cohort are needed. In this study we focused on identifying miRNAs regulated differently in tumor tissue with neighboring healthy tissue. While this comparison might be used for prediction of treatment outcome, its value as a biomarker for cancer diagnosis is limited. Further analysis of miRNA expression should be performed on patients’ serum and circulating tumor cells in patients’ plasma, which could be used as a routine diagnostic tool.

## 5. Conclusions

In this study, we conducted analysis of the expression of several miRNAs in tumor and healthy tissue collected from patients diagnosed with HNSCC in the oral cavity, oropharynx, and larynx regions. Our results demonstrated that tumor tissue differs significantly in expression of miRNAs from healthy tissue and that the tumors of different locations differed in expression of some miRNAs. Additionally, the expression of miR-449-5p was the strongest predictor of overall survival in oral cancer, with several other miRNAs showing some correlation with survival. Analysis of combined expression of miRNAs presented two groups of miRNAs (miR6510-3p and miR-34c-5p for oral cancer, and miR449a-5p, miR-6510-3p, and miR-133a-5p for laryngeal cancer), which showed a very strong ability to distinguish between tumor tissue and neighboring healthy tissue. Future research on miRNAs should focus on an in-depth investigation of their mechanisms of action in HNSCC. Specifically, the mechanism related to the effects of miR-6510-3p expression in this tumor should be investigated, since its role in HNSCC has not yet been described.

## Figures and Tables

**Table 1:** Clinical characteristics of the study group.

	Tumor Location		
Characteristic	Oral Cavity	Oropharynx	Larynx
Number of subjects	37	9	33
Age (years, mean ± SD)	59.3 (±11.2)	52.9 (±12.1)	62.2 (±12.5)
Sex, n (%)			
Male	30 (81%)	5 (56%)	25 (76%)
Female	7 (19%)	4 (44%)	8 (24%)
T stage, n (%)			
T1-2	26 (70%)	6 (67%)	4 (12%)
T3-4	11 (30%)	3 (33%)	28 (85%)
NA	0 (0%)	0 (0%)	1 (3%)
N stage, n (%)			
N0	11 (30%)	0 (0%)	14 (42%)
N+	25 (68%)	8 (89%)	17 (52%)
NA	1 (3%)	1 (11%)	2 (6%)
M stage, n (%)			
M0	37 (100%)	9 (100%)	30 (91%)
NA	0 (0%)	0 (0%)	3 (9%)
Grading, n (%)			
G1	6 (16%)	0 (0%)	2 (6%)
G2	27 (73%)	2 (22%)	24 (73%)
G2/G3	1 (3%)	0 (0%)	1 (3%)
G3	3 (8%)	4 (44%)	5 (15%)
NA	0 (0%)	3 (33%)	1 (3%)

SD—standard deviation; T stage—size of tumor; N stage—spread to lymph nodes; M—distant metastasis; NA—data not available.

## Supplementary Materials

The following are available online at https://www.mdpi.com/article/10.3390/cancers13163980/s1, Figure S1: Boxplots showing the comparison of expression of 12 miRNAs between tumor tissue and nearby healthy tissue in the oral cavity, oropharynx, and larynx, Figure S2: ROC curves showing accuracy of gene expression (−∆∆Ct) of each miRNA as a biomarker in distinguishing oral tumor tissue from nearby healthy tissue, Figure S3: ROC curves showing accuracy of gene expression (−∆∆Ct) of each miRNA as a biomarker in distinguishing oropharyngeal tumor tissue from nearby healthy tissue, Figure S4: ROC curves showing accuracy of gene expression (−∆∆Ct) of each miRNA as a biomarker in distinguishing laryngeal tumor tissue from nearby healthy tissue, Figure S5: Kaplan Meier survival curves showing comparison of overall survival between low and high miRNA expression de-fined by optimal cutoff of −∆∆Ct in oral cancer, Figure S6: Kaplan Meier survival curves showing comparison of overall survival between low and high miRNA expression defined by optimal cutoff of −∆∆Ct in oropharyngeal cancer, Figure S7: Kaplan Meier survival curves showing comparison of overall survival between low and high miRNA expression defined by optimal cutoff of −∆∆Ct in laryngeal cancer, Table S1: Stem-loops used for miRNA expression analysis, Table S2: Median expression of miRNAs in tumor tissue relative to healthy tissue. Values calculated using −∆∆Ct method, Table S3: Summary of multiple Cox regression analysis investigating association of the combined miRNA expression (miRNAs detected in combined biomarker analysis) with overall survival of oral cancer and larynx cancer with age at diagnosis, T staging and N staging under control.
