# Supplementary material for: miRNAs as Biomarkers for Diagnosing and Predicting Survival of Head and Neck Squamous Cell Carcinoma Patients
Source: Cancers (Basel). 2021 Aug 6;13(16):3980. doi: 10.3390/cancers13163980 (PMC8392400; doi:10.3390/cancers13163980)
Supplement: Supplementary file 1 [file cancers-13-03980-s001.zip › cancers-1311559-supplementary.pdf]

cancers

MDPI

Article

miRNAs as biomarkers for diagnosing and predicting survival of head and neck squamous cell carcinoma patients

Igor Piotrowski [1,2,3], Xiang Zhu [3], Tatiana Dandolini Saccon [4], Sarah Ashiqueali [3], Augusto Schneider [5], Allancer Divino de Carvalho Nunes [3], Sarah Noureddine [3], Agnieszka Sobecka [1,6], Wojciech Barczak [1,6], Mateusz Szewczyk [6,7], Wojciech Golusiński [6,7], Michal M Masternak [3,6] and Paweł Golusiński [8,9] *

[1] Radiobiology Lab, Department of Medical Physics, Greater Poland Cancer Centre, 61-866 Poznan, Poland; igor.piotrowski@wco.pl
[2] Department of Electroradiology, Poznan University of Medical Sciences, ul. Garbary 15, 61-866 Poznan, Poland.
[3] Burnett School of Biomedical Sciences, College of Medicine, University of Central Florida, Orlando, Florida, USA.
[4] Centro de Desenvolvimento Tecnológico, Universidade Federal de Pelotas, Pelotas, RS, Brazil.
[5] Faculdade de Nutrição, Universidade Federal de Pelotas, Pelotas, RS, Brazil.
[6] Department of Head and Neck Surgery, Poznan University of Medical Sciences, Poznan, Poland.
[7] Department of Head and Neck Surgery, Greater Poland Cancer Centre, Poznan, Poland.
[8] Department of Otolaryngology and Maxillofacial Surgery, University of Zielona Gora, Zielona Gora, Poland; p.golusinski@cm.uz.zgora.pl
[9] Department of Maxillofacial Surgery, Poznan University of Medical Sciences, Poznan, Poland.

* Correspondence: p.golusinski@cm.uz.zgora.pl

Supplementary Materials:

Table S1: Stem-loops used for miRNA expression analysis.

miRNA	Mature miRNA sequence	Stem-loop sequence	miRBase Accession Number
hsa-miR-146a-5p	UGAGAACUGAAU-UCCAUGGGUU	CCGAUGUGUAUCCUCAGCUUUGAGAACUGAAU-UCCAUGGGUUGUGUCAGUGUCAGACCU-CUGAAAUUCAGUUCUUCAGCUGGGAUAUCUCUGUCAUCGU	MIMAT0000449
hsa-miR-449a	UGGCAGUG-UAUUGUUAGCUGGU	CUGUGUGUGAUGAGCUGGCAGUG-UAUUGUUAGCUGGUUGAAUAUGUGAAUGGCAU-CGGCU-AACAUGCAACUGCUGUCUUAUUGCAUAUACA	MIMAT0001541
hsa-miR-126-5p	CAUUAUUACU-UUUGGUACGCG	CGCUGGCGACGGGACAUUAUUACU-UUUGGUACGCGCUGUGACACU-UCAAACUCGUACCGUGA-GUAAUAAUGCGCCGUCCACGGCA	MIMAT0000444
hsa-miR-34a-5p	UGGCAGUG-UCUUAGCUGGUUGU	GGCCAGCUGUGAGUGUUUCUUUGGCAGUG-UCUUAGCUGGUUGUUGUGAGCAAUAGUAAGGAA-GCAAUCAGCAAGUAUACUGCCCUAGAAGUGCUGCACGUUGUGGGGCCC	MIMAT0000255
hsa-miR-34b-5p	UAGGCAGUGUCAU-UAGCUGAUUG	GUGCUCGGUUUGUAGGCAGUGUCAUUAGCUGAU-UGUACUGUGGUGGUUACAAUCACU-AACUCCACUGCCAUCAAAACAAGGCAC	MIMAT0000685

hsa-miR-<br>34c-5p	AGGCAGUG-<br>UAGUUAGCUGAU-<br>UGC	AGUCUAGUUACUAGGCAGUGUAGUUAGCUGAU-<br>UGCUAAUAGUACCAAUCACUAACCACAC-<br>GGCCAGGUAAAAAGAUU	MIMAT0000686
hsa-miR-<br>217-5p	UACUGCAUCAG-<br>GAACUGAUUGGA	AGUAUAAUUAUUACAUAGUUUUUGAUGUCG-<br>CAGAUACUGCAUCAGGAACUGAUUGGAU-<br>AAGAAUCAGUCACCAUCAGUUCCUAAUGCAUUGC<br>CUUCAGCAUCUAAACAAG	MIMAT0000274
hsa-miR-<br>378c	ACUGGACUUG-<br>GAGUCAGAA-<br>GAGUGG	GGAGGCCAUCACUGGACUUGGAGUCAGAAGAGUG-<br>GAGUCGGGUCAGACUUCAACUCUGACU-<br>UUGAAGGUGGUGAGUGCCUC	MIMAT0016847
hsa-miR-<br>6510-3p	CACCGACU-<br>CUGUCUCCUGCAG	AGCAGCAGGGGAGAGAGAGGAGUCCUCUAGACAC-<br>CGACUCUGUCUCCUGCAGAU	MIMAT0025477
hsa-miR-<br>96-5p	UUUGGCACUAG-<br>CACAUUUUUGCU	UGGCCGAUUUUGGCACUAGCACAU-<br>UUUUGCUUGUGUCUCUCCGCUCUGAGCAAUCAU-<br>GUGCAGUGCCAAUAUGGGAAA	MIMAT0000095
hsa-miR-<br>149-5p	UCUGGCUCCGUG-<br>UCUUCACUCCC	GCCGGCGCCCGAGCUCUGGCUCCGUG-<br>UCUUCACUCCCGUGCUUGUCCGAG-<br>GAGGGAGGGAGGGACGGGGGCUGUGCUGGGGCAG<br>CUGGA	MIMAT0000450
hsa-miR-<br>133a-5p	AGCUGGUAAAAUG-<br>GAACCAAAU	ACAAUGCUUUGCUAGAGCUGGUAAAAUGGAAC-<br>CAAAUCGCCUCUUCAAUGGAU-<br>UUGGUCCCCUUCAACCAGCUGUAGCUAUGCAUUG<br>A	MIMAT0026478

Table S2: Median expression of miRNAs in tumor tissue relative to healthy tissue. Values calculated using -ΔΔCt method.

miRNA	Tumor location		
	Oral cavity	Oropharynx	Larynx
miR-146a-5p	0.76	0.86	1.27
miR-449a-5p	1.99	3.04	3.36
miR-126-5p	-0.98	-1.67	-0.41
miR-34a-5p	-0.17	-0.66	0.92
miR-34b-5p	1.41	1.32	2.07
miR-34c-5p	1.06	2.45	1.83
miR-217-5p	0.98	-0.32	0.60
miR-378c-5p	-2.08	-0.21	-1.00
miR-6510-3p	-4.60	-3.97	-1.81
miR-96-5p	0.30	-2.50	0.86
miR-149-5p	-0.94	-3.94	0.04
miR-133a-5p	-0.96	-1.73	-15.58

Table S3: Summary of multiple Cox regression analysis investigating association of the combined miRNA expression (miRNAs detected in combined biomarker analysis) with overall survival of oral cancer and larynx cancer with age at diagnosis, T staging and N staging under control.

Factor	HR(SE)	P-value
Oral cancer		
Age at diagnosis	0.986(0.039)	0.726
miR6510	0.942(0.184)	0.760
miR34c_5p	1.2(0.317)	0.491
T-staging		
T1-2	1(reference)	
T2-3	6.962(8.76)	0.123
T3-4	1.635(1.391)	0.564
N-staging		
N0	1(reference)	
N+	4.701(5.359)	0.174
Laryngeal cancer		
Age at diagnosis	1.014(0.032)	0.654
miR449a	0.816(0.107)	0.121
miR6510	1.031(0.154)	0.839
miR149	0.846(0.214)	0.508
T-staging		
T1-2	1(reference)	
T3-4	0.416(0.368)	0.322
N-staging		
N0	1(reference)	
N+	3.159(2.191)	0.097

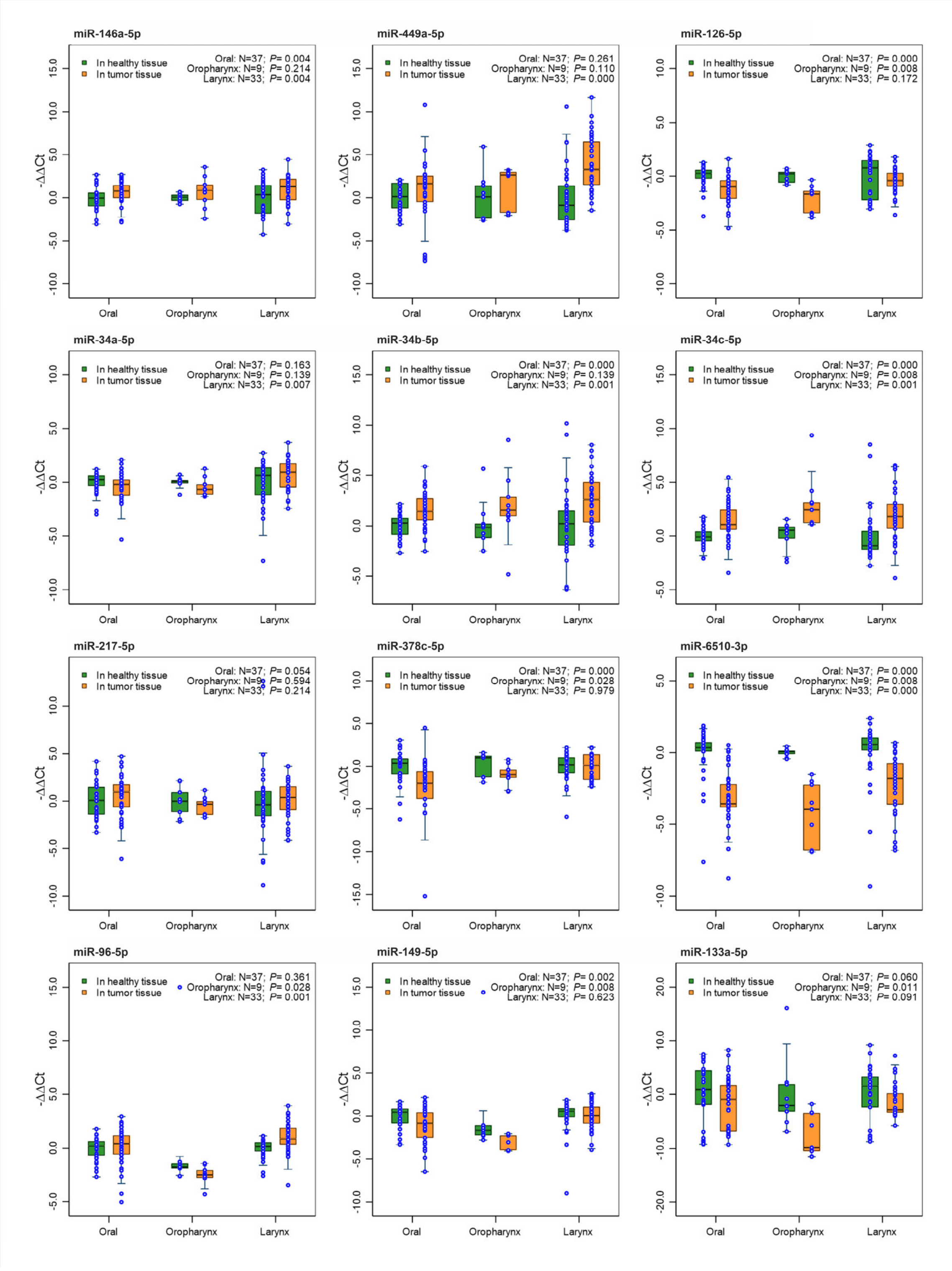

Figure S1. Boxplots showing the comparison of expression of 12 miRNAs between tumor tissue and nearby healthy tissue in the oral cavity, oropharynx, and larynx. Expression was calculated using -ΔΔCt method; values above 0 denote upregulation, and values below 0 denote downregulation of miRNA, compared with healthy tissue. Wilcoxon signed-rank test was performed.

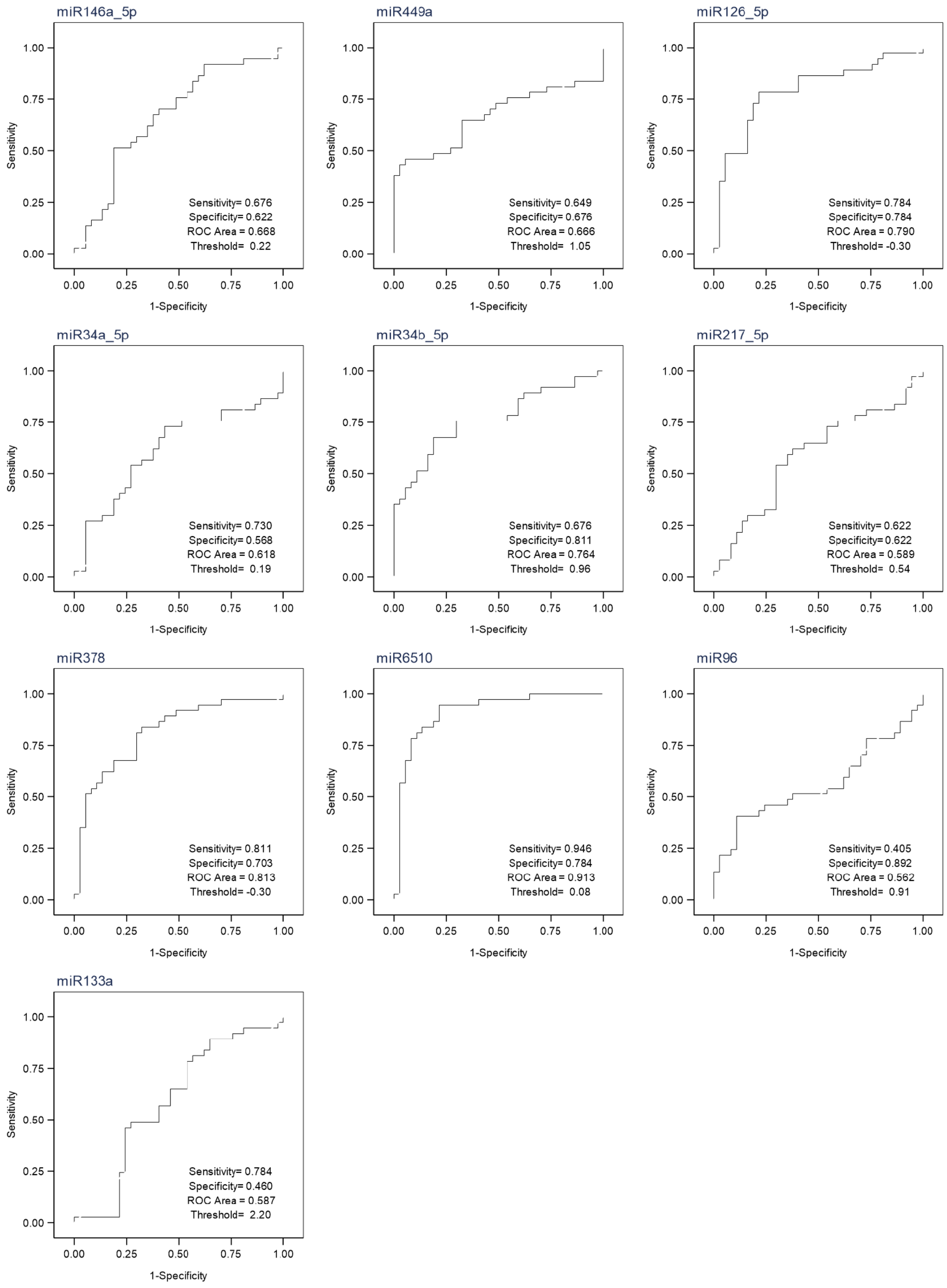

Figure S2. ROC curves showing accuracy of gene expression (-ΔΔCt) of each miRNA as a biomarker in distinguishing oral tumor tissue from nearby healthy tissue.

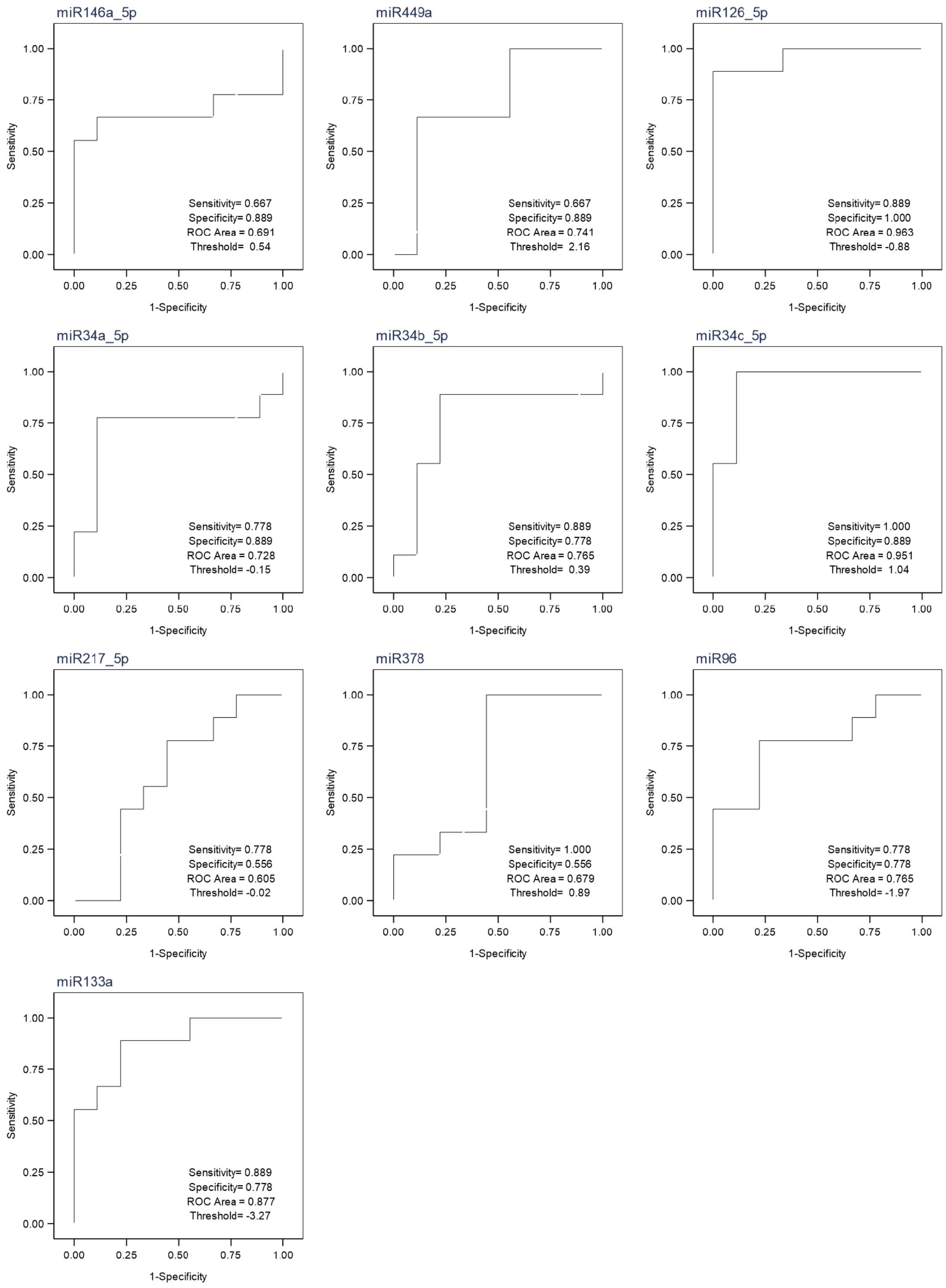

Figure S3. ROC curves showing accuracy of gene expression (-ΔΔCt) of each miRNA as a biomarker in distinguishing oropharyngeal tumor tissue from nearby healthy tissue.

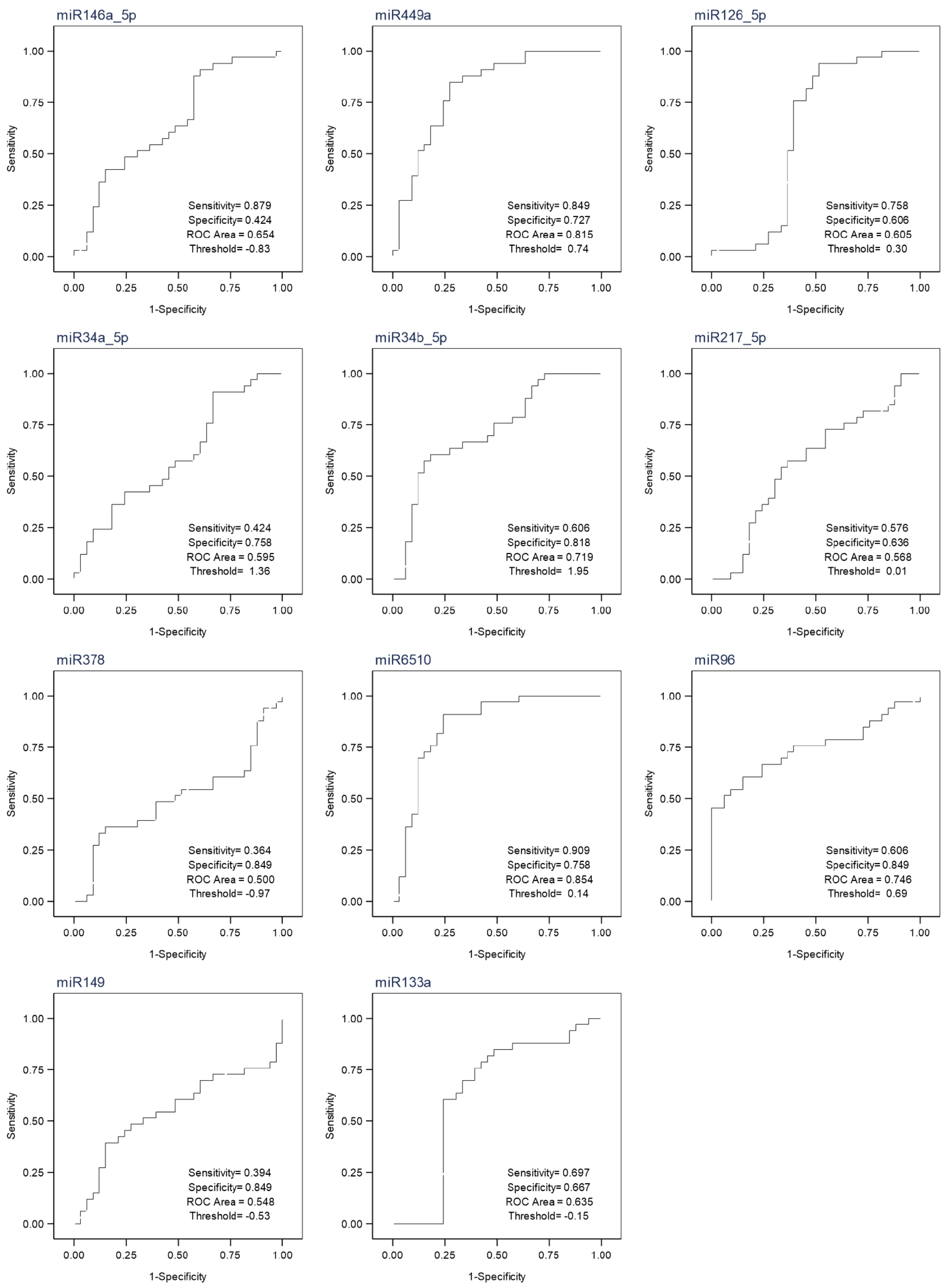

Figure S4. ROC curves showing accuracy of gene expression (-ΔΔCt) of each miRNA as a biomarker in distinguishing laryngeal tumor tissue from nearby healthy tissue.

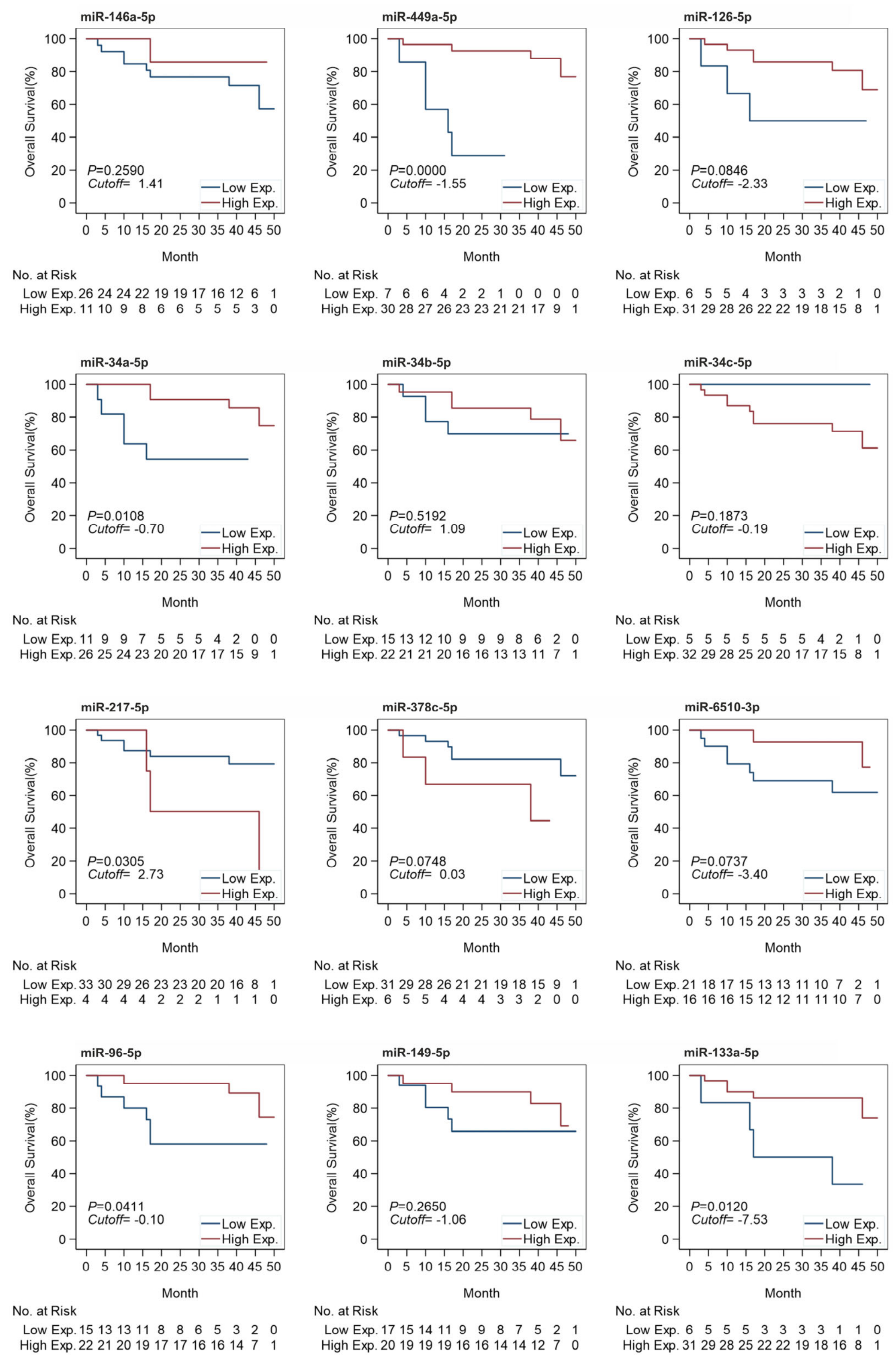

Figure S5. Kaplan Meier survival curves showing comparison of overall survival between low and high miRNA expression defined by optimal cutoff of -ΔΔCt in oral cancer.

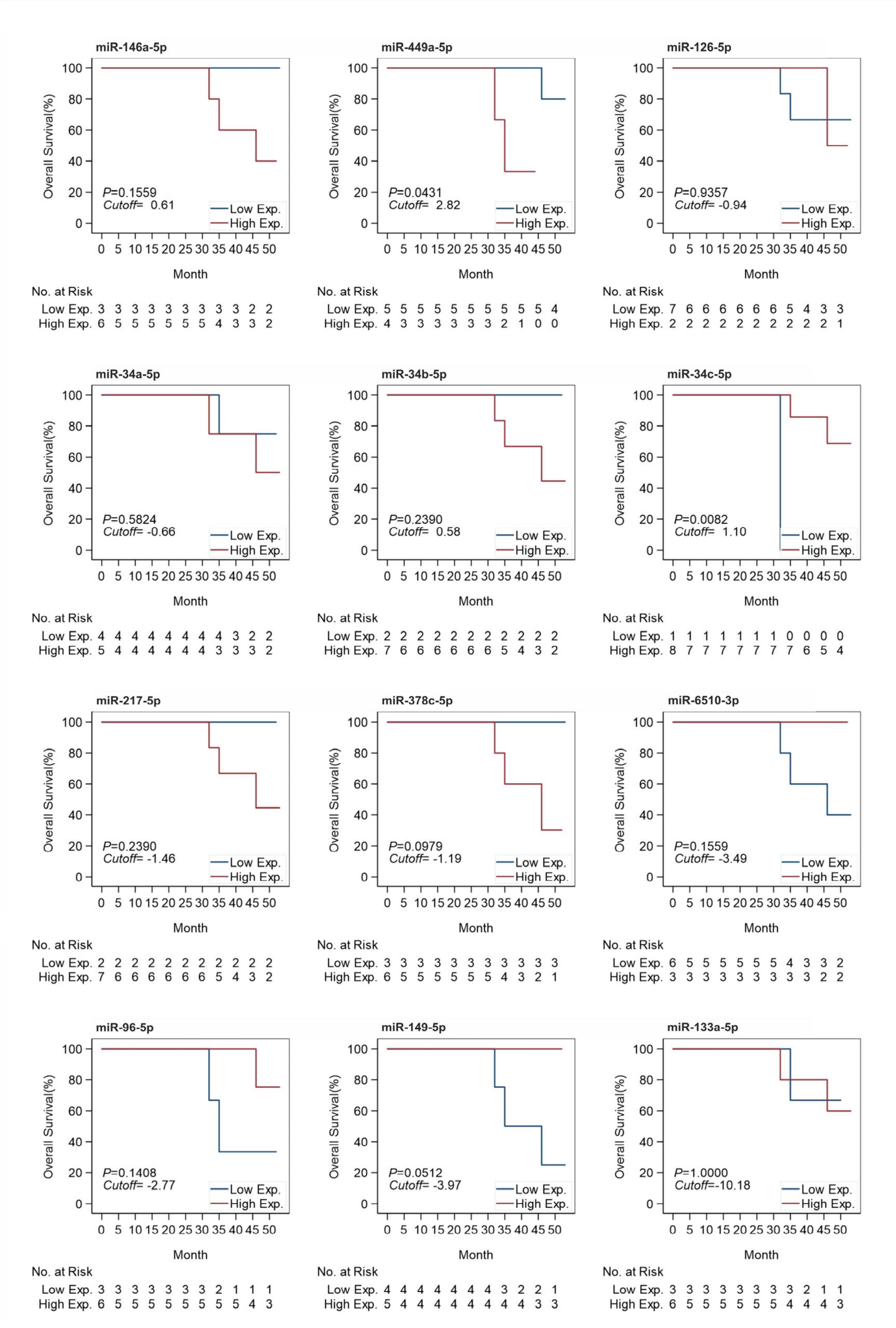

Figure S6. Kaplan Meier survival curves showing comparison of overall survival between low and high miRNA expression defined by optimal cutoff of -ΔΔCt in oropharyngeal cancer.

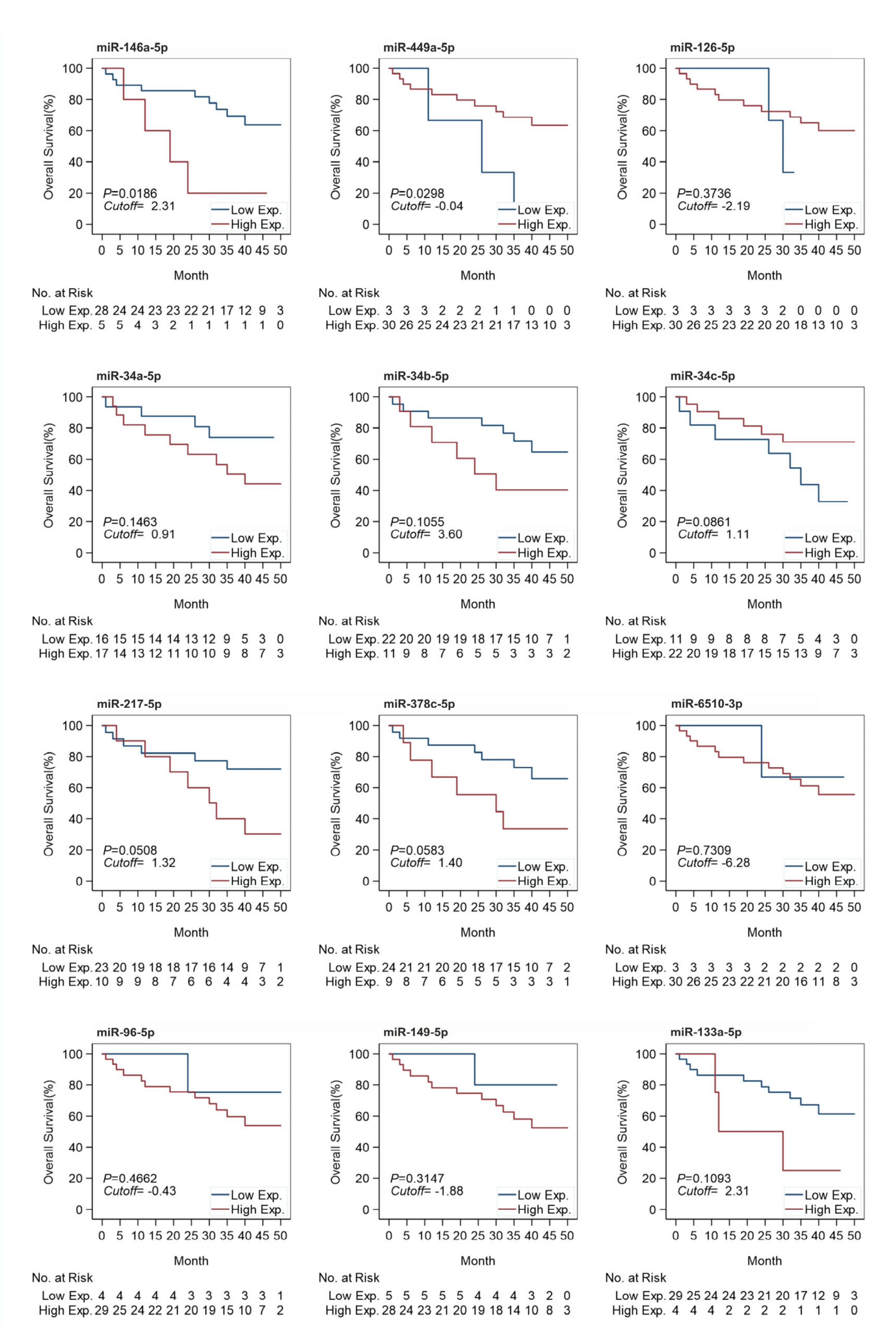

Figure S7. Kaplan Meier survival curves showing comparison of overall survival between low and high miRNA expression defined by optimal cutoff of -ΔΔCt in laryngeal cancer.
